# Quantitative quadruple-label immunofluorescence of mitochondrial and cytoplasmic proteins in single neurons from human midbrain tissue

**DOI:** 10.1016/j.jneumeth.2014.05.026

**Published:** 2014-07-30

**Authors:** Anne Grünewald, Nichola Z. Lax, Mariana C. Rocha, Amy K. Reeve, Philippa D. Hepplewhite, Karolina A. Rygiel, Robert W. Taylor, Doug M. Turnbull

**Affiliations:** a Wellcome Trust Centre for Mitochondrial Research, Institute of Ageing and Health, Newcastle University, Newcastle upon Tyne, UK; b Institute of Neurogenetics, University of Lübeck, Lübeck, Germany

**Keywords:** Mitochondria, Complex I, Complex IV, Single cell analysis, Midbrain neurons, Immunofluorescent labelling

## Abstract

**Background:**

Respiratory chain (RC) deficiencies are found in primary mtDNA diseases. Focal RC defects are also associated with ageing and neurodegenerative disorders, e.g. in substantia nigra (SN) neurons from Parkinson's disease patients. In mitochondrial disease and ageing, mtDNA mutational loads vary considerably between neurons necessitating single cell-based assessment of RC deficiencies. Evaluating the full extent of RC deficiency within SN neurons is challenging because their size precludes investigations in serial sections. We developed an assay to measure RC abnormalities in individual SN neurons using quadruple immunofluorescence.

**New method:**

Using antibodies against subunits of complex I (CI) and IV, porin and tyrosine hydroxylase together with IgG subtype-specific fluorescent labelled secondary antibodies, we quantified the expression of CI and CIV compared to mitochondrial mass in dopaminergic neurons. CI:porin and CIV:porin ratios were determined relative to a standard control.

**Results:**

Quantification of expression of complex subunits in midbrain sections from patients with mtDNA disease and known RC deficiencies consistently showed reduced CI:porin and/or CIV:porin ratios.

**Comparison with existing method(s):**

The standard histochemical method to investigate mitochondrial dysfunction, the cytochrome *c* oxidase/succinate dehydrogenase assay, measures CIV and CII activities. To also study CI in a patient, immunohistology in additional sections, i.e. in different neurons, is required. Our method allows correlation of the expression of CI, CIV and mitochondrial mass at a single cell level.

**Conclusion:**

Quantitative quadruple-label immunofluorescence is a reliable tool to measure RC deficiencies in individual neurons that will enable new insights in the molecular mechanisms underlying inherited and acquired mitochondrial dysfunction.

## Highlights

• We developed an assay to quantify respiratory chain deficiencies in single neurons.
• Quadruple-label immunofluorescence was combined with quantitative image analysis.
• The single-cell assay was applied to tyrosine hydroxylase-positive midbrain neurons.
• The expression of complexes I and IV was determined relative to mitochondrial mass.
• The assay proved specific in patients with known respiratory chain deficiencies.

## 1 Introduction

The oxidative phosphorylation (OXPHOS) system comprises approximately 85 polypeptides. These polypeptides assemble (i) four respiratory chain (RC) complexes, complex I (CI) to complex IV (CIV), (ii) one complex facilitating adenosine triphosphate (ATP) generation, complex V (CV), and (iii) two mobile electron carriers, coenzyme Q (CoQ) and cytochrome *c* (Mitchell, 1961). The OXPHOS system is situated in the lipid bilayer of the inner mitochondrial membrane (Hatefi, 1985; Saraste, 1999). The passage of electrons from CI or CII via CIII and CIV catalyzes the transfer of protons from the mitochondrial matrix across the inner mitochondrial membrane to the inter-membrane space building an electrochemical gradient. This gradient is the driving force for CV, ATP synthase, to generate ATP from ADP and inorganic phosphate (Hatefi, 1985; Saraste, 1999).

RC enzyme deficiency, in particular involving CI and CIV (Greaves et al., 2010; Reeve et al., 2008; Swalwell et al., 2011), is present in patients with primary mitochondrial diseases (DiMauro and Schon, 2003). In addition, there is increasing evidence for a role of RC enzyme deficiency in ageing and neurodegenerative diseases such as Parkinson's disease (PD) (Bender et al., 2006; Langston et al., 1983; Schapira et al., 1989). In primary mitochondrial DNA (mtDNA) diseases, dysfunction of RC enzyme complexes is due to inherited mutations in the mitochondrial genome with the level of heteroplasmy (mutational burden) varying considerably between cells (Taylor and Turnbull, 2005). In PD, and normal human ageing, an accelerated accumulation of somatic mtDNA deletions in substantia nigra neurons has been reported (Bender et al., 2006; Kraytsberg et al., 2004). As a consequence of varying mutational loads, cells differ in the degree of OXPHOS deficiency which necessitates analysis on a single cell level to determine the nature of the biochemical defect. In many tissues (e.g. skeletal muscle, large intestine), information concerning RC activities and expression levels can be obtained from sequential sections (Campbell et al., 2013; Greaves et al., 2010; Rowan et al., 2012). In the CNS, however, investigations concerning RC enzyme function and abundance have so far been performed in populations rather than single cells, since the size of most neuron types, including the dopaminergic neurons of the substantia nigra, precludes serial studies.

To enable us to simultaneously explore the degree of deficiency of CI and CIV (both of which have mtDNA-encoded subunits) in single dopaminergic neurons from postmortem midbrain tissue, we have established a quantitative immunofluorescent protocol for quadruple labelling.

## 2 Materials and methods

### 2.1 Human tissue

Skeletal muscle tissue was obtained from an elderly control, following informed consent, with age-related RC deficiencies (age: 78, sex: female) and snap frozen in isopentane at −160 °C. Muscle was cyrosectioned (10 μm) and sections allowed to air dry for 60 min at room temperature (RT) before immunohistology or histochemistry was performed.

Brain tissue was provided by the Newcastle Brain Tissue Resource (NBTR) with ethical approval. To optimize the quadruple labelling immunofluorecence protocol, post-mortem brain samples were obtained from two patients with mitochondrial disease exhibiting CI and CIV deficiencies, one idiopathic Parkinson disease patient (age: 77, sex: female) and one control with no neurological phenotype (age: 55, sex: male). The mitochondrial disease patients included one individual with MERRF due to the m.8344A>G mutation (age: 58, sex: male) and one patient with Kearns Sayre syndrome (KSS) due to a single, large-scale mtDNA deletion (age: 40, sex: female) (Reeve et al., 2013). Sections of formalin-fixed paraffin-embedded midbrain blocks were cut at 5 μm using a microtome (Microm International) and mounted on to SuperFrostTM slides (Thermo Fisher Scientific).

### 2.2 Immunofluoresence labelling and histochemistry with muscle sections

For immunofluorescent staining, snap frozen muscle sections were dried, fixed in 4% (w/v) paraformaldehyde (PFA) for 10 min and then washed three times for 2 min in Tris-buffered saline containing 1% Tween-20 (TBST). Sections were then blocked in TBST and 1% (v/v) normal goat serum (NGS) for 1 h at RT. Monoclonal mouse antibodies against complex I-20 (NDUFB8, IgG subtype 1, Abcam, ab110242), and CIV-1 (COX1, IgG subtype 2a, Abcam, ab14705) were diluted 1:100 in 1% NGS and incubated over night at 4 °C. To remove unbound primary antibodies, sections were washed three times in TBST for 5 min. Then IgG subtype-specific secondary anti-mouse antibodies conjugated to Alexa fluor 488 and 546 (both Life Technologies, A21131 and A21143, respectively) were applied (1:100 in 1% NGS TBST) for 60 min at RT. Again, sections were washed three times for 5 min in TBST. To quench autofluorescence, Sudan black (0.3% w/v in 70% v/v ethanol) solution was applied for 10 min followed by three short washes in TBST. Finally, sections were mounted in Prolong Gold Antifade Reagent (Life Technologies).

For immunohistology with chromogens, air-dried muscle sections were fixed in 4% PFA for 10 min, followed by rinsing in TBST for 10 min. The sections were permeabilized in a graded (70%, 95%, 100%, 100%, 95%, 70% v/v, 10 min each) methanol series. Endogenous peroxidase activity was quenched by addition of hydrogen peroxide 0.3% (v/v) to 95% (v/v) methanol. Sections were rinsed in TBST and blocked in 5% (w/v) BSA TBST for 30 min at RT. Next, sections were incubated overnight with anti-CI-20 and CIV-1 antibodies (1:100 in 5% (w/v) BSA TBST) at 4 °C. Following three washes in TBST, sections were treated with the MenaPath kit according to manufacturer's protocol (Menarini Diagnostics). After five washes in TBST for 5 min, peroxidase activity was detected by incubation in 3,3′-diaminobenzadine tetrahydrochloride solution (SigmaFast DAB tablets dissolved in distilled water) for 5 min at RT.

CIV (COX) histochemical activity in muscle was detected using a published technique (Johnson et al., 1988).

### 2.3 Immunofluorescence with midbrain sections

To deparaffinize and rehydrate, paraffin-fixed midbrain sections were incubated at 60 °C for 30 min. This was followed by sequential washing steps in Histoclear (2 times for 5 min) (National Diagnostics), and a graded ethanol series (100%, 100%, 95%, 70% v/v, 5 min each). Next, sections were washed in distilled water. Antigen retrieval was performed in 1 mM EDTA, pH 8, in a pressure cooker for 40 min followed by another wash in distilled water and a short wash in TBST. Sections were blocked in TBST and 1% (v/v) NGS for 1 h at RT. Monoclonal mouse antibodies against CI-20, CIV-1, the mitochondrial mass marker porin (IgG subtype 2b, Abcam, ab14734) and a rabbit polyclonal antibody directed against the dopaminergic marker tyrosine hydroxylase (TH, Sigma, T8700) were used at a concentration of 1:100. Midbrain sections were incubated in the primary antibody dilution overnight at 4 °C. This was followed by three wash steps for 5 min in TBST. For quadruple immunofluorescent labelling, midbrain sections were incubated with IgG subtype-specific secondary anti-mouse antibodies conjugated with Alexa Fluor 488, 546 or 647 (A21240) and a secondary anti-rabbit Alexa Fluor 405 (A31556) antibody (all Life Technologies, 1:100) for 1 h at RT. Next, sections were washed 3 times for 5 min in TBST and then incubated in Sudan black solution for 10 min. This was followed by three washes in TBST and mounting in Prolong Gold (Life Technologies).

### 2.4 Fluorescence microscopy imaging and densitometric analysis

Imaging of midbrain sections was performed using the epifluorescence Axio Imager M1 (Zeiss) microscope. Fluorophores of secondary antibodies were chosen so that cross-talk, i.e. overlap between their absorption and emission spectra, was minimized. Single-label controls were used to determine the remaining fraction of “bleed-through” between different filters and the lower signal threshold was adjusted accordingly during imaging. To further avoid cross-talk, the fluorophore with longest peak emission wavelength was imaged first. Dopaminergic neurons were identified by their neuromelanin deposits and TH-positive staining. In acquired images, all detectable dopaminergic neurons were analyzed independent of cellular morphology or protein abundance of CI-20, CIV-1 or porin up to a total cell number of 100. For densitometric analysis of the target proteins, Image J Software (version 1.46r) was used. Images from the four channels were imported as an image sequence. The background signal for all antibodies was determined in a non-stained area. In each channel, the threshold was adjusted according to the background signal and kept constant between different sections for each individual. Cells were outlined manually according to their TH signal. Within these defined areas, mean signal intensities of all channels were determined simultaneously using the “analyze stack” function in Image J.

Images of sequential muscle sections were also acquired using the Axio Imager M1 (Zeiss).

### 2.5 Statistical analysis

Prism version 4.01 and the non-parametric Mann–Whitney test were used to compare intensities between individuals as the obtained densitometric data from cellular analysis were not normally distributed.

## 3 Results

### 3.1 Detection of RC enzyme activities and protein levels in serial muscle sections from a mitochondrial disease case

**Fig. 1:**
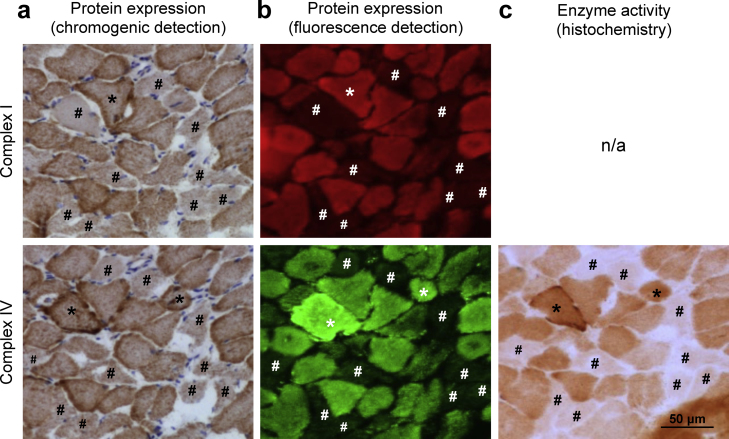
Correlation of respiratory chain complex expression and enzyme activity in serial skeletal muscle sections from a control with age-related RC deficiencies. (a and b) Immunohistological detection of CI-20 and CIV-1 with chromogens or fluorescence labelling. Increased (#) or decreased (*) immunoreactivity of CI-20 or CIV-1 in individual muscle fibres of the mitochondrial disease patient is detectable in sequential sections independent of the visualization method used. (c) Cytochrome *c* oxidase (COX) histochemistry. COX activity corresponds to CIV-1 expression in individual muscle fibres of the control individual, confirming good correlation between the two different techniques.

Serial sections of skeletal muscle from an elderly control subject with age-related RC deficiencies were employed to test the compatibility of two immunohistology approaches – use of (i) chromogens or (ii) fluorescence labelling for visualization of target proteins. Both assays were applied to sequential muscle sections to investigate the expression of CI and CIV. We chose to examine subunit 1 of CIV, as expression levels had been reported to correlate with activity in this complex (Mahad et al., 2009). Furthermore, an antibody against subunit 20 of CI was employed which had previously been shown to reliably detect deficiencies of RC CI in tissue from mtDNA disease patients (Reeve et al., 2013). Both immunohistology approaches identified identical fibres as CI and/or CIV-deficient independent of the immunohistology method used. A small number of fibres also showed increased levels of CI and/or CIV expression (Fig. 1a and b).

For CIV, a histochemical technique allows measurement of cytochrome *c* oxidase (COX) activity in tissue samples. The method uses 3,3′-diaminobenzidine (DAB) which is oxidized by functional CIV, forming a brown pigment. Consequently, cells with dysfunctional CIV will remain free of the DAB oxidation product. In concordance with previous studies (Mahad et al., 2009; Tanji and Bonilla, 2008), COX activity corresponded well with CIV-1 expression (Fig. 1c), indicating loss of activity as a result of loss of the catalytic subunit of CIV in some of the muscle fibres. In the case of CI, a direct correlation of protein expression and activity on single cell level is not possible as there is currently no available protocol to quantify NADH:ubiquinone oxidoreductase histochemical activity reliably.

### 3.2 Quadruple immunofluorescence and expression analysis of mitochondrial proteins at a single neuron level in midbrain sections

In contrast to muscle fibres, midbrain neurons commonly do not reach beyond the section thickness of 5 μm. Information on the expression of multiple RC enzyme complexes in an individual neuron can, therefore, not be obtained from the analysis of sequential sections. Due to this limitation and following the observation of a link between CIV activity and expression, we established an immunohistology protocol which allows simultaneous detection of CI-20 and CIV-1 normalized to mitochondrial mass in dopaminergic neurons. As a mitochondrial marker, the highly abundant ion channel forming protein porin (which spans the outer mitochondrial membrane) was used (Colombini, 1980). Porin is a nuclear-encoded mitochondrial protein which is preserved in the presence of mtDNA disease (Blachly-Dyson et al., 1994). An antibody directed against tyrosine hydroxylase which catalyzes the rate limiting step in dopamine synthesis (Flatmark and Stevens, 1999) was used to identify dopaminergic neurons.

The main hurdle with the investigation of proteins in brain sections by means of fluorescent immunohistology is the interference of autofluorescence with the target signal. This autofluorescence is, on the one hand, caused by lipofuscin pigment which is present at high concentrations in postmitotic cells such as neurons. On the other hand, autofluorescence can be fixative-induced since aldehydes react with proteins generating fluorescent products (Billinton and Knight, 2001). To counteract autofluorescence, we incubated the midbrain sections in Sudan black as a final step before image analysis (Romijn et al., 1999).

**Fig. 2:**
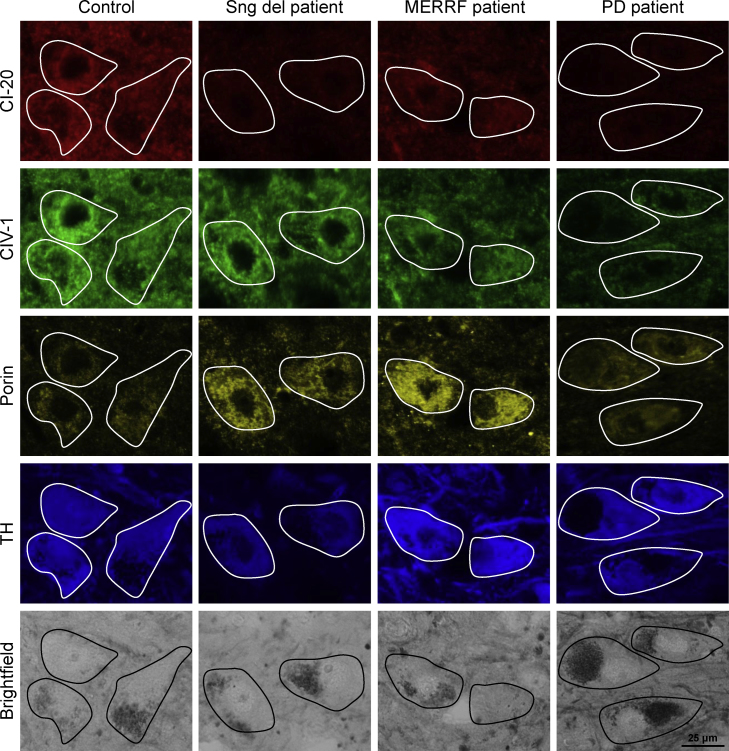
Fluorescence labelling immunohistology of complex I-20, complex IV-1, porin and tyrosine hydroxylase (TH) in dopaminergic midbrain neurons of an age-matched control, patients with mitochondrial disease and PD. Epifluorescence microscopy images of the employed four fluorescence channels (emission wavelengths: 405 nm, 488 nm, 546 nm and 647 nm) were taken with exposure times that were kept constant across the different investigated midbrain sections. Images indicate differences in protein expression of respiratory chain enzyme subunits and mitochondrial mass between patients and control. Taking porin levels into account, the single large-scale mtDNA deletion patient showed isolated CI deficiency, whereas the m.8344A>G MERRF and the PD patients showed combined loss of CI and CIV expression. Positive TH staining and neuromelanin deposits (dark areas in brightfield channel) point towards the dopaminergic character of the investigated neurons. The outlines of TH-positive neurons are shown in all channels. MERRF – myoclonic epilepsy and ragged-red fibre disease, PD – Parkinson disease, Sng del – single large-scale mtDNA deletion.

To test the specificity of our protocol, we used midbrain sections from a patient with a single large-scale mtDNA deletion, a patient with the m.8344A>G mutation causing MERRF, a PD patient and an elderly neurologically normal control individual. CI-20 and CIV-1 expression had previously been studied in populations of dopaminergic neurons in the substantia nigra of both mitochondrial disease patients (cases “MERRF2” and “Single deletion 1” in Reeve et al. (2013)). This analysis revealed 44% of completely CI-20-deficient and only 1% of completely CIV-1-deficient cells in the single mtDNA deletion case. In the m.8344A>G MERRF patient, 22% of dopaminergic midbrain neurons were completely deficient of CI-20 and 23% were completely deficient of CIV-1 (Reeve et al., 2013). The quadruple-label immunofluoresence images also showed isolated CI-20 deficiency for the single large-scale mtDNA deletion patient and combined CI-20 and CIV-1 deficiency for the m.8344A>G patient (Fig. 2). In comparison, microscopy images of midbrain sections of the PD patient indicated a joint loss of CI-20 and CIV-1 with a slight trend towards CI deficiency (Fig. 2, right panel). In addition, respiratory chain enzyme deficiencies coincided with elevated porin levels in MERRF disease patient neurons consistent with evidence of increased mitochondrial biogenesis.

**Fig. 3:**
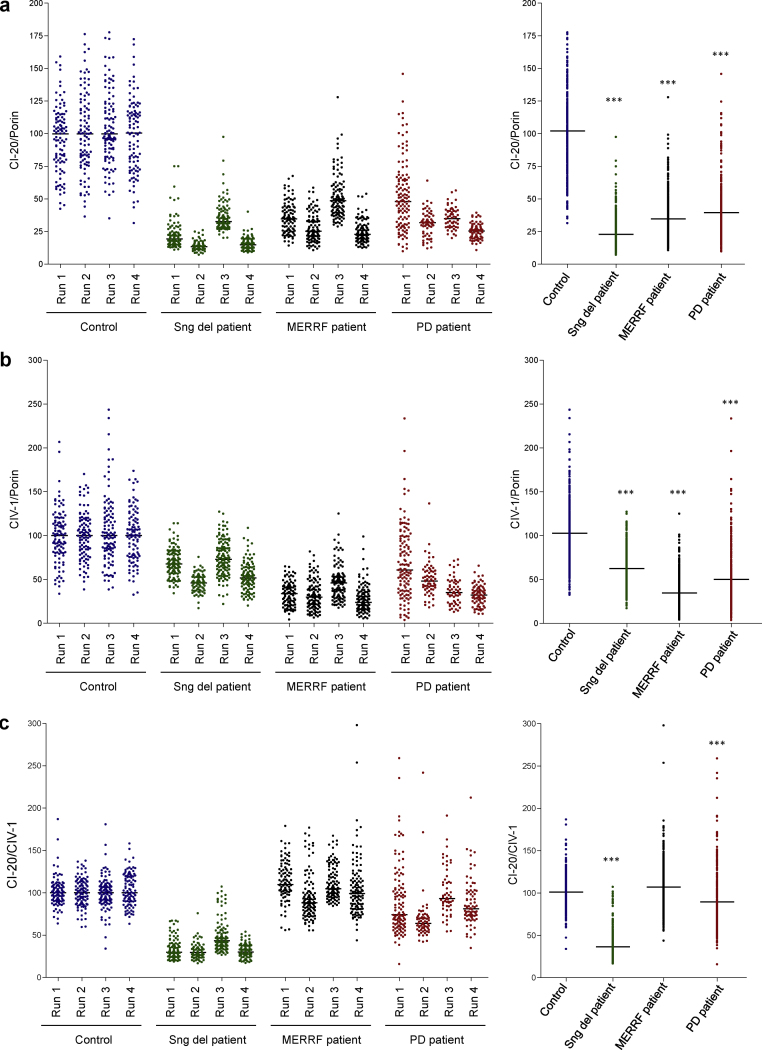
Densitometry analysis of complex I-20 and complex IV-1 expression in dopaminergic midbrain neurons from one control individual, and patients with mitochondrial disease or PD. Data from consecutive experiments is shown in the left panel and the combined results per person are given in the right panel. (a and b) The protein levels of CI-20 and CIV-1 of individual neurons were expressed relative to the mitochondrial mass marker porin. This analysis consistently showed lower CI-20 than CIV-1 levels in single large-scale mtDNA deletion patient neurons when compared to control neurons. By contrast, a patient with m.8344A>G MERRF presented with a combined loss of CI and CIV expression in all analyzed midbrain sections. Similarly, in a PD patient, reduced CI-20 and CIV-1 levels were detected in all individual runs. (c) Calculation of the CI-20 to CIV-1 ratio showed a clear shift towards CI deficiency only in the neurons of the single large-scale mtDNA deletion patient. Protein levels were determined in 50–100 neurons per section and four sections per individual from four independent experimental runs were analyzed. Tyrosine hydroxylase (TH) staining was used to identify dopaminergic neurons. The control ratio was set to 100% in each run serving as internal standard. Dots indicate ratios per individual cell and the median of each dataset is marked as a line. MERRF – myoclonic epilepsy and ragged-red fibre disease, PD – Parkinson disease, Sng del – single large-scale mtDNA deletion. *p < 0.05; ***p < 0.001.

To quantify the expression of CI-20, CIV-1 and porin, we established an image analysis protocol in Image J. After background correction, pixel intensities within areas of interest (defined by the outlines of TH-positive neurons) were determined. The CI-20:porin, CIV-1:porin and CI-20:CIV-1 ratios were calculated for approximately 100 neurons in the substantia nigra of a control brain section. Median ratios for the control neurons were determined and set to 100%. The expression ratios of all individual cells (from patients and the control) were expressed relative to the control median. This approach was repeated three times using different midbrain sections from the same individuals. In each experiment, CI expression in the single, large-scale mtDNA deletion patient (who was found to be more severely affected than the m.8344A>G MERRF patient according to Reeve et al. (2013)) was indeed lower than in the m.8344A>G MERRF patient (Fig. 3a, left panel). The total median ratios derived from the combined data of all experimental runs supported this observation (CI-20:porin: control, 100%; single large-scale mtDNA deletion, 19%, *p* < 0.001; m.8344A>G MERRF, 33%, *p* < 0.001; PD, 32%, *p* < 0.001) (Fig. 3a, right panel). The CIV-1:porin ratios also showed deficiency but the findings in the different patients varied from the CI deficiency. Taking all runs together, we determined a median CIV-1:porin ratio of 60% (*p* < 0.001) for the single large-scale mtDNA deletion patient, of 32% (*p* < 0.001) for the m.8344A>G MERRF patient and 42% (*p* < 0.001) for the PD patient (Fig. 3b). For the mitochondrial disease patients, this finding was again consistent with previous data from cell population analyses (Reeve et al., 2013). When the CI-20:CIV-1 ratios were plotted, a marked shift towards CI deficiency was observed for the single mtDNA deletion patient with 67% (*p* < 0.001) less CI-20 than CIV-1. In case of the PD patient, this ratio indicated 23% (*p* < 0.001) less CI-20 than CIV-1. By contrast, the m.8344A>G MERRF patient showed a CI-20:CIV-1 similar to the control (Fig. 3c). These quantitative findings reflected the impressions obtained from visual inspection of the immunolabeled midbrain sections (Figs. 2 and 3).

## 4 Discussion

In primary mtDNA disease, different mtDNA mutations and mutation heteroplasmy levels cause great variability in the degree of mitochondrial dysfunction in different cell populations. Under these circumstances functional analyses in single cells are preferred over investigations in tissue homogenates. The standard approach to investigate mitochondrial function on single cell level is the COX/SDH assay (Old and Johnson, 1989). This histochemical method gives information about the activity of RC complexes IV and II with CII activity serving as a marker of mitochondrial mass. In contrast, there is currently no histochemical protocol available which allows accurate determination of CI (rotenone sensitive NADH:ubiquinone oxidoreductase) activity in individual cells in tissue sections. However, like CIV, CI deficiency has been reported in PD (Schapira et al., 1989). To study CI expression in tissue, immunohistology with chromogens is commonly used. This method is limited to the application of one antibody at the time. Consequently, the expression of RC complex subunits cannot be normalized for mitochondrial abundance and correlations between different proteins on single cell level are precluded. Therefore, to determine the degree of deficiency of CI and to investigate the relationship between CI and CIV in single neurons, immunofluorescence labelling is required.

When comparing CIV histochemistry and immunohistology readouts from consecutive muscle sections, we found, in concordance with earlier studies, that CIV activity and expression correlated in the majority of fibres (Koob et al., 2012; Siskova et al., 2010). Since there is no histochemical assay to reliably detect CI activity in addition to CIV activity, we established a protocol that allows simultaneous detection and expression quantification of four target proteins in individual neurons from postmortem midbrain tissue. We quantified the protein levels of CI-20, CIV-1 and mitochondrial porin in neurons that expressed tyrosine hydroxylase as a marker of dopaminergic signalling. To verify the assay, midbrain sections from mitochondrial disease patients with known CI and/or CIV deficiencies were subjected to the protocol. We consistently identified the single large-scale mtDNA deletion patient as the mitochondrial disease case with most severe CI deficiency and the m.8334A>G MERRF patient as the case with most severe CIV deficiency. These results are in line with previous findings using chromogens (Reeve et al., 2013). The observed variation in level of deficiency is likely due to the use of different midbrain sections from the same individual in different experimental runs. Interestingly, respiratory chain enzyme deficiencies were accompanied by an increase in mitochondrial mass in mtDNA disease patient neurons resulting in very low Cx:porin ratios. We hypothesize that additional mitochondria are generated through biogenesis to compensate for the reduced OXPHOS capacity in these cells. However, these new mitochondria also lack functional respiratory chain complexes as indicated by low CI:porin (and CIV:porin) ratios. By contrast, in the PD patient SN neurons only a slight increase in mitochondrial mass was detected. In cellular models of PD, impaired mitochondrial maintenance has been identified as the underlying pathological mechanism (de Vries and Przedborski, 2013). The observed changes in CI, CIV and porin levels may therefore be a result of reduced mitochondrial turnover by autophagy.

In summary, we established quadruple immunofluorescence together with densitometric imaging analysis as tools to quantify protein levels of subunits of multiple RC enzyme complexes in single neurons. Applying our method, the relationship between RC complexes can be studied in discrete cell types in the brain with availability of cell type-specific antibodies being the only limitation. This proves particularly useful when investigating the molecular causes of mitochondrial diseases or neurodegenerative disorders which can both affect various brain regions. Using quadruple immunofluorescence in combination with LCM and DNA analysis, (i) RC enzyme deficiencies may further be ascribed to a specific genetic defect in the mtDNA and (ii) the heteroplasmy threshold for developing a RC defect can be determined within a single cell.

## Financial disclosures

The funders had no role in study design, data collection and analysis, decision to publish, or preparation of the manuscript.
